# Extraction, characterization of polyphenols from certain medicinal plants and evaluation of their antioxidant, antitumor, antidiabetic, antimicrobial properties, and potential use in human nutrition

**DOI:** 10.3389/fnut.2023.1125106

**Published:** 2023-06-21

**Authors:** Abdul Mueed, Sahar Shibli, Diana A. Al-Quwaie, Mada F. Ashkan, Mona Alharbi, Humidah Alanazi, Najat Binothman, Majidah Aljadani, Kamlah Ali Majrashi, Mashael Huwaikem, Mohammed A. S. Abourehab, Sameh A. Korma, Mohamed T. El-Saadony

**Affiliations:** ^1^State Key Laboratory of Food Science and Technology, Nanchang University, Nanchang, Jiangxi, China; ^2^Department of Food Technology, Institute of Food and Nutrition, Arid Agriculture University, Rawalpindi, Pakistan; ^3^Food Science Research Institute, National Agricultural Research Centre, Islamabad, Pakistan; ^4^Biological Sciences Department, College of Science & Arts, King Abdulaziz University, Rabigh, Saudi Arabia; ^5^Department of Biochemistry, College of Science, King Saud University, Riyadh, Saudi Arabia; ^6^Department of Chemistry, College of Sciences & Arts, King Abdulaziz University, Rabigh, Saudi Arabia; ^7^Cinical Nutrition Department, College of Applied Medical Sciences, King Faisal University, Al Ahsa, Saudi Arabia; ^8^Department of Pharmaceutics, College of Pharmacy, Umm Al-Qura University, Makkah, Saudi Arabia; ^9^Department of Pharmaceutics and Industrial Pharmacy, College of Pharmacy, Minia University, Minia, Egypt; ^10^Department of Food Science, Faculty of Agriculture, Zagazig University, Zagazig, Egypt; ^11^Department of Agricultural Microbiology, Faculty of Agriculture, Zagazig University, Zagazig, Egypt

**Keywords:** medicinal plants, polyphenols, antioxidant, antidiabetic, antitumor, antimicrobial, human nutrition

## Abstract

**Introduction:**

Dietary medicinal plants are among the most sought-after topics in alternative medicine today due to their preventive and healing properties against many diseases.

**Aim:**

This study aimed to extract and determine the polyphenols from indigenous plants extracts, i.e., *Mentha longifolia, M. arvensis, Tinospora cordifolia, Cymbopogon citratus, Foeniculum vulgare, Cassia absus, Camellia sinensis, Trachyspermum ammi*, *C. sinensis* and *M. arvensis*, then evaluate the antioxidant, cytotoxicity, and antimicrobial properties, besides enzyme inhibition of isolated polyphenols.

**Methods:**

The antioxidant activity was evaluated by DPPH, Superoxide radical, Hydroxyl radical (OH^.^), and Nitric oxide (NO^.^) scavenging activity; the antidiabetic activity was evaluated by enzymatic methods, and anticancer activity using MTT assay, while the antibacterial activity.

**Results:**

The results showed that tested medicinal plants’ polyphenolic extracts (MPPE) exhibited the most significant antioxidant activity in DPPH, hydroxyl, nitric oxide, and superoxide radical scavenging methods because of the considerable amounts of total polyphenol and flavonoid contents. UHPLC profile showed twenty-five polyphenol complexes in eight medicinal plant extracts, categorized into phenolic acids, flavonoids, and alkaloids. The main polyphenol was 3-Feroylquinic acid (1,302 mg/L), also found in *M. longifolia*, C. *absus*, and *C. sinensis*, has a higher phenolic content, i.e., rosmarinic acid, vanillic acid, chlorogenic acid, p-coumaric acid, ferulic acid, gallic acid, catechin, luteolin, 7-*O*-neohesperideside, quercetin 3,7-*O*-glucoside, hesperidin, rutin, quercetin, and caffeine in the range of (560–780 mg/L). At the same time, other compounds are of medium content (99–312 mg/L). The phenolics in *C. sinensis* were 20–116% more abundant than those in *M. longifolia*, *C. absus*, and other medicinal plants. While *T. cordifolia* is rich in alkaloids, *T. ammi* has a lower content. The MTT assay against Caco-2 cells showed that polyphenolic extracts of *T. ammi* and *C. citratus* had maximum cytotoxicity. While *M. arvensis, C. sinensis*, and *F. vulgare* extracts showed significant enzyme inhibition activity, *C. sinensis* showed minor inhibition activity against α-amylase. Furthermore, *F. vulgare* and *C. sinensis* polyphenolic extracts showed considerable antibacterial activity against *S. aureus, B. cereus, E. coli*, and *S. enterica*.

**Discussion:**

The principal component analysis demonstrated clear separation among medicinal plants’ extracts based on their functional properties. These findings prove the therapeutic effectiveness of indigenous plants and highlight their importance as natural reserves of phytogenic compounds with untapped potential that needs to be discovered through advanced analytical methods.

## 1. Introduction

Dietary and medicinal plants have gained the special attention of researchers today due to their abundance of functional compounds such as polyphenols, flavonoids, proteins, and vitamins in different plant parts (1, 2). These polyphenols are secondary metabolites that play an essential role in maintaining human health by acting as antioxidants, anticancer, and antimicrobial agents and are thus increasingly being utilized in allopathic medicine. The bioactivity of these substances is attributed to their aromatic rings, which carry hydroxyl groups at diverse positions (1, 3).

Although they have some drawbacks related to instability, less bioavailability, oxidation and degradation by light, heat, and less membrane permeability (4), a variety of essential techniques are utilized to increase the medicinal benefits of polyphenols by combining them with macromolecules. Direct polymerization of polyphenol monomers or combination with macromolecules makes polyphenols more stable. Among the strategies for direct polymerization, they included free radical, and step-growth, enzyme-catalyzed procedures. Polyphenols, such as catechin, quercetin, epicatechin, EGCG, rutin, tannic acid, and ferulic acid, have generated polymers with molecular weights between 890 and 77,000 (5). Polymerization maintains or enhances the antioxidant activity of polyphenols, and various possible medical uses have been established, such as inhibition of XO, proteinase, and LDL oxidation; medication transport; and cancer, antibacterial, and antifungal therapies. By esterification, amidation, free radical grafting, and enzyme-assisted grafting, polyphenol polymer conjugates have been manufactured (6). Various polyphenols, i.e., gallic acid, ferulic acid, caffeic acid, tannic acid, EGCG, catechin, quercetin, curcumin, and epicatechin, have been conjugated with synthetic and natural polymers, such as dextran, gelatin, inulin, chitosan, hyaluronic acid, and alginate (7).

As with direct polymerization, the conjugation of polyphenols to polymers maintains or enhances antioxidant activity. Polyphenol polymers are characterized by several beneficial therapeutic uses such as bone regeneration, skin care, Alzheimer’s disease, diabetes, LDL oxidation, and enzyme inhibition; antioxidant, anticancer, antibacterial, and anti-inflammatory therapies (8). Due to the resistance of microbes to many conventional medicines or their ineffectiveness against oxidative stress or various cancers, in this study, the medicinal plants’ polyphenolic extract were used as antioxidants and antimicrobials, as they have many diverse mechanisms through which they mitigate microbial resistance or free radicals (9–13).

The antioxidant activity of polyphenol complexes plays a role in preventing different diseases such as respiratory, diabetes, neurodegenerative, cancer, and cardiovascular illnesses (3, 14, 15). Plants possess significant antimicrobial, antifungal, and antiviral activity due to their phenolic compounds, as a result of which they are being used to manufacture many pharmaceutical preparations due to their harmless nature, multifunctionality, and effectiveness against resistant microbes (16–20). Furthermore, the anticancer activity of these medicinal plants against malignant cell lines and their synergistic action with various cancer drugs have also been revealed in recent investigations (21, 22). These valuable functions are attributable to flavonoids and phenolic acids; flavonoids account for approximately two-thirds of the dietary polyphenols in plant-based foods and play a valuable role in human health. However, phenolic acids represent one-third. The scientific community is becoming more interested in these phenolic acids because of their antioxidant properties and anticipated health advantages (23). The phenolic acids are widespread in fruits, coffee, spices, and vegetables consumed daily by humans representing around 25 mg per day (24).

In this study, eight dietary and medicinal plants (*Mentha longifolia, Mentha arvensis, Tinospora cordifolia, Cymbopogon citratus, Foeniculum vulgare, Cassia absus, Camellia sinensis*, and *Trachyspermum ammi*) were selected based on their ethnomedicinal value and composition of bioactive compounds. These plants have been used for a long time as a remedy for common diseases such as fever, pain, stomach disorders, obesity, appetite loss, wounds, and indigestion. The monomers of polyphenolic polymers in these plants are well known, such as menthone, butyl octanol, menthol, isomenthol, and eucalyptol were identified in extracts of *M. longifolia*, and *M. arvensis* extracts (25, 26), tinosporide, tinosporine, magnoflorine, giloinsterol, giloin in *T. cordifolia* (27), limonene, camphene, farnesol, cyclic monoterpene in *C. citratus* (28), estragole, α-phellandrene, trans-anethole, fenchone in *F. vulgare* (29), chaksine, chrysophanol, aloe-emodin in *C. absus* (30), catechins, caffeine, epigallocatechin gallate, in *C. sinensis* (31), thymol, p-cymene, myrcene, carvacrol in *T. ammi* extract (32). These plants are used as antiemetic, antidiabetic, anti-inflammatory, anticancer, antimicrobial, diuretic, and inhibitors of pancreatic and adipocyte enzymes in conventional medicinal plants. In a study by Şen, Akbulut (33), male genital diseases can be treated by *Galium palustre*, *Pilosella leucopsilon* subsp. *pilisquama*, and *Astragalus nitens*. Also, *Tilia rubra* subsp. *caucasica*, can treat disorders in the respiratory and digestive systems and skin illnesses (34). In Turkey, Güler, Polat (35), 65 indigenous medicinal plants from 38 families have been identified as treatments for animal ailments. Additionally, 48 traditional plants belonging to 29 families in the region proved their use in curing stomach and respiratory disorders, besides pain relief (36, 37).

As No previous studies shed light on the isolation and identification of polyphenol polymers in eight medicinal plants, and limited published data on some plants, such as *C. absus, T. cordifolia*, and *C. citratus* health benefits; Similarly, data on the toxicity and antidiabetic activity of most vegetal extracts’ are scarce in the literature. Therefore, the primary purpose of this study was to isolate and investigate the polyphenol polymers from eight medicinal plants extracts by UHPLC, then evaluate the biological activity of these eight plant extracts through phytochemical analysis, antioxidant, cytotoxic, and antibacterial, antidiabetic assays.

## 2. Materials and methods

### 2.1. Chemicals

α-Glucosidase, α-amylase, and acarbose, 2,2-diphenyl-1-picrylhydrazyl (DPPH), were from Sigma-Aldrich (St. Louis, MO, USA), naphthyl ethylenediamine dihydrochloride, gallic acid, quercetin, sodium nitrate, methanol, trisaminomethane (Tris), nitro blue tetrazolium chloride (NBT), 1,4 Dihydronicotinamide adenine dinucleotide (NADH), phenazine methosulfate (PMS), FeSO_4_, saffron, salicylic acid, sodium nitroprusside, sulfanilic acid reagent, glacial acetic acid, and naphthyl ethylene diamine dihydrochloride were acquired from Sigma-Aldrich (Shanghai, China), 3-(4,5-dimethylthaizol-2-yl)-2,5-diphenytetrazolium bromide (MTT) was procured from Enzo Life Science (Plymouth Meeting, PA, USA). D-Hank’s buffer, dimethyl sulfoxide (cell-culture grade), and 0.25% EDTA trypsin were obtained from (Solarbio, China); RPMI 1640 medium and fetal bovine serum were acquired from (GIBCO, USA). Microbial strains; *Escherichia Coli* ATCC 8739, *Staphylococcus aureus* ATCC 25923, *Bacillus cereus* ATCC 10876, and *Salmonella enterica* ATCC 14028 were obtained from Microbiologic (St. Cloud, Minnesota, USA).

### 2.2. Collection of medicinal plants and their ethnobotanical/ethnomedicine survey

Data on the therapeutic uses of various plants were gathered by interviewing local people and conventional medicine practitioners. Plant information was obtained, including their local names, parts of the plant, and medicinal uses, as indicated in Table 1. Eight plants, namely, *M. arvensis* (Voucher no. RAW 101886), *F. vulgare* (Voucher no. AM-4), *T. ammi* (Voucher no. AM-5), *M. longifolia* (Voucher no. RAW 101885), *C. absus* (Voucher no. AM-3), *T. cordifolia* (Voucher no. RAW 101884), *C. citratus* (Voucher no. raw material (RAW) 101887), *C. sinensis* (Voucher no. AM-2), were selected in the present study based on their ethnobotanical significance and their botanical identity were authenticated from National Herbarium, Bio-resources Conservation Institute, National Agricultural Research Center, Islamabad, Pakistan through voucher numbers mentioned above. The plant samples were collected from various areas of Khyber Pakhtunkhwa (Swabi, Mardan, and Hazara) and stored at room temperature in plastic bags until analysis.

**TABLE 1:** The taxonomy of selected dietary medicinal plants and their nutritional and medical uses.

Scientific name/local name	Family	Plants’ part	(%) Yield	Medicinal uses	Mechanism	References
*Cassia absus* (Chasku)	Fabaceae	Seed	21	Used as a blood tonic and astringent	Helpful in treating asthma and tumors; Seeds, leaves, and roots are used for skin infection, leucoderma, cathartic, syphilis, ophthalmia, and constipation	(30)
*Foeniculum vulgare* (Sounf)	Umbelliferae	Seed	20	Mouth freshener increases appetite, relieves indigestion, dysentery, gastrointestinal and genitourinary ailments	Carminative, Panacea, hypo-lipidemic, galactagogue, anti-aging, anti-spasmodic, antiallergic, antipyretic, antipolitics, anti-hirsutism, antinociceptive, anti-inflammatory, antiviral, anti-mutagenic, anti-microbial, anti-thrombotic, anxiolytic, diuretic, Chemo-modulatory action, hypoglycemic, cytoprotective and antitumor, estrogenic properties, expectorant, memory-enhancing property, hepatoprotective and oculohypotensive activities	(29)
*Mentha Arvensis* (Podina*)*	Lamiaceae	Stem and leaves	24	Good tonic improves digestion, helpful in diarrhea, stomach disorders, sinusitis, headache, chest congestion, colds, influenza, and sore throat	It treats spleen disease, jaundice, and asthma. The leaf extract is excellent in treating indigestion, rheumatism, infantile problems, vomiting during pregnancy, and hysteria. Anti-pruritic, anti-septic, stimulant, anesthetic in treating dermatological cases, diuretic, thermogenic dentrific, anodyne, antispasmodic, antihelminthic, cardiotonic, vulnerary, expectorant, deodorant, sudorific, febrifuge, digestive, contraceptive, carminative, and anti-hepatalgic.	(139)
*Tinospora Cordifolia* (Gillo)	Menispermaceae	Stem	23	Decoction of the stem is a common household tonic to cure fevers, emetic, and dyspepsia; also, a decoction of gillo and saunf is used for the animal’s fever and appetite growth	Viral hepatitis, gonorrhea, anemia, urinary disease, and dysentery	(27)
*Trachyspermun ammi* (Ajwain)	Apiaceae	Seed	22	Effective in gastric troubles, stomach disorders, nausea, reflux, vomiting, abdominal cramps, loss of appetite, cough, leukoderma, and pleurisy	For Dysphonia, paralysis, tremor, palsy, and other neural disorders, anthelmintic medicine and used as an antidote for various natural toxic agents, diuretic agents, aphrodisiacs, carminatives, and a galactagogue	(32)
*Cymbopogon citratus (Lemon grass)*	Poaceae	Leaves	25	Solves obesity, coughing, fever, hypocholesteremia, flu, urination problems, hemoptysis, gastric and sudorific problems	Treats backache, sprain, asthma, headaches, and pneumonia; antispasmodic, emmenagogue, antitussive, antiseptic, anti-rheumatic stimulating, digestive, effective against colds, analgesic, anti-allergic, anti-cardiopathic, cure for urinary ducts inflammation, diuretic, anti-thermic, and diaphoretic	(28)
*Camellia sinensis (Tea leaves)*	Theaceae	Leaves	25	Boiled tea leaves with lemon extract are a common household tonic to cure colds, respiratory disorders, and flu	Reduce chronic diseases such as allergies, anxiety, depression, and insomnia; enhance heart function; improve the immune system; aid in digestion, and headaches; strengthen teeth and bones, intestinal disorders; fights virus, and high blood pressure	(31)
*Mentha longifolia ((Velany) jangli podina)*	Lamiaceae	Stem and leaves	19	Relieve vomiting, cough, a stomach infection, diarrhea, and fever	Antimicrobial, insecticidal, antiplatelet, and antispasmodic properties, and stimulates digestion. Cures aerophagia, viral hepatitis, colitis, and gastric acidity	(151)

### 2.3. Preparation of plant material

The parts of medicinal plants in Table 1 were carefully washed with sterilized distilled water to eliminate any dirt before being utilized in the experiment. After that, the two-step drying procedure was carried out. In the first step, the samples were shade dried at a moderate temperature (21–30°C) for two weeks by spreading them out on paper and rolling them back and forth to let the most moisture evaporate. In the second step, the shed-dried plants were dehydrated in an oven (Model-600, Memmert, Germany) at 45°C for three hours to remove any remaining moisture. A dry mill (Kenwood Multi-Mill, Havant, UK) was used to grind plant samples into a fine powder which was then passed through a screen (mesh size 30) to ensure an equal pulverization of the plants. The samples were stored in sterilized, sealed plastic bags at room temperature until use (38).

### 2.4. Extraction of medicinal plants’ polyphenolic extracts (MPPE)

Ten grams from each medicinal plant’s powder was extracted with 200 mL of 80% methanol for 1 h in an ultrasonic bath (Transsonic-700, 37 kHz, Germany) at 50°C. The MPPE was centrifuged at 3,000 rpm for 10 min; the supernatant was obtained, and the residues were discarded. The solvent in the supernatant was evaporated using an R-200-Buchi-rotary evaporator (Switzerland), and the concentrated MPPE was obtained; The yield percentage of MPPE was evaluated using the following equation, the methanol-free extract was lyophilized and retained in water when the use (39).

$$Percentageyieldofextract=\frac{Weightofextract}{Weightofsample}\times 100$$

### 2.5. Qualitative analysis of polyphenol polymers in MPPE

The MPPE were subjected to qualitative analysis of phytogenic compounds for checking the presence or absence of compounds such as alkaloids, carbohydrates, flavonoids, saponins, phenols, tannins, steroids, terpenoids, and glycosides using various methods, as shown in Table 2.

**TABLE 2:** Qualitative polyphenol polymers analysis of MPPE.

Phytochemicals	Test	*Trachyspermum ammi*	*Foeniculum vulgare*	*Cassia absus*	*Camellia sinensis*	*Mentha longifolia*	*Mentha arvensis*	*Tinospora cordifolia*	*Cymbopogon citratus*
Alkaloids	Mayer’s test (152)	+	+	+	+	+	+	+	+
Carbohydrates	Molisch’s test (153)	+	+	+	+	+	+	+	−
Saponins	Frothing test (154)	−	−	+	−	+	+	+	+
Flavonoids	Alkaline reagent test (155)	+	+	+	+	+	+	+	+
Phenol	Ferric chloride test (156)	+	+	+	+	+	+	+	+
Tannins	Gelatin test (152)	+	+	+	+	+	+	+	+
Steroids	Mandelin’s test (157)	+	+	+	+	−	+	+	−
Terpenoids	Lieberman Burchardt test (157) and Salkowski test (158)	+	+	−	+	+	−	+	+
Glycosides	Keller-Kiliani test (152)	−	−	+	+	+	+	−	−

*n* = 3 (+) positive results for the phytochemicals; (-) negative results for the phytochemicals.

### 2.6. Determination of the total phenolic content (TPC) in MPPE

The TPC in MPPE was determined using the Folin-Ciocalteu method following Singleton and Rossi (40) using gallic acid as a standard. In brief, 1 mL of each MPPE was mixed with 2.5 mL of a 10% Folin-Ciocalteu reagent, then 2.5 mL of a 7.5% Na_2_CO_3_ solution. The mix was kept in a dark place at room temperature; the Agilent 8453 UV-vis spectrophotometer (USA) was used to measure the generated color at 765 nm. to estimate the quantity of polyphenol, the standard curve of methanolic gallic acid (12.5, 25, 50, 100, 200, and 400 μg mL^–1^) was utilized. TPC was measured as gallic acid equivalents (mg g^–1^ extract).

### 2.7. Evaluation of the total flavonoid content (TFC) in MPPE

The TFC of MPPE was evaluated by the AlCl_3_ colorimetric method using quercetin as a standard (41). 1 mL of MPPE was diluted with 10 mL of quercetin, 4 mL of distilled water, and 0.3 mL of NaNO_3_ (5%) and then mixed well. Then, 0.3 mL of AlCl_3_ (10%) was added after 5 min. Finally, 2 mL of 1M NaOH was added after 6 min, and the final volume was adjusted to 10 mL with distilled water. The absorbance of the obtained color was read at 415 nm. The quantitative assessment was based on a standard calibration curve of various concentrations of methanolic quercetin (62.5, 125, 250, and 500 g mL^–1^). The TPC of MPPE was calculated as mg QE g^–1^.

### 2.8. Polyphenol complex UHPLC profile of MPPE

The polyphenol complex of eight MPPE (*Mentha longifolia, Mentha arvensis, Tinospora cordifolia, Cymbopogon citratus, Foeniculum vulgare, Cassia absus, Camellia sinensis*, and *Trachyspermum ammi*) was identified by Ultra-HPLC system (Thermo Fisher Scientific, USA). Separation column with 150 × 2.0 mm dimensions, 5 μm (Gemini C18-110, Phenomenex, USA) was used. The mobile phases were (A) Formic acid in water 0.1% and (B) in acetonitrile 0.1%. The flow rate was adjusted to 0.2 mL/min, and the column was heated to 30°C. The MPPE was dissolved in a methanol and water mix (50:50, *v*: *v*). the sample size was 10 μL flow through the separation column. The data MS was detected by negative ionization techniques in the 100- and 1,400 m/z range.

### 2.9. Antioxidant activity of MPPE

#### 2.9.1. DPPH free radical scavenging activity

The DPPH* scavenging capacity of MPPE was evaluated by Brand-Williams et al. (42) with slight modifications. The control of DPPH was prepared by dissolving 24 μg of DPPH in 100 mL of methanol. 1 mL of different concentrations of MPPE (25, 100, 200, 400 μL mL^–1^) were mixed with 3 mL of DPPH and kept for fifteen minutes in a dark place; then the absorbance was measured at 517 nm. The inhibition of DPPH* was calculated using the following equation.

$$\%\mathrm{Inhibition}\mathrm{of}\mathrm{DPPH}\mathrm{radical}=\frac{Blankabsorbance-Sampleabsorbance}{Blankabsorbance}\times 100$$

#### 2.9.2. Superoxide radical scavenging activity

The superoxide radical scavenging capacity of MPPE was determined using Nishikimi et al. (43) method with slight modification. 1 mL of MPPE concentrations (50–400 μg mL^–1^) was mixed with 4.5 mL of Tris-HCl buffer solution (16 mM, pH 8.0), 0.5 mL of 250 μM nitro blue tetrazolium chloride (NBT) solution, 0.5 mL of 400 μM nicotinamide adenine dinucleotide (NADH) and 0.5 mL of 50 μM phenazine methosulfate (PMS). The mixture was incubated for 5 min at room temperature, and its absorbance was recorded at 560 nm compared to a blank sample. The superoxide radical scavenging capacity was calculated using the following formula.

$$\%\mathrm{Inhibition}\mathrm{of}\mathrm{superoxide}\mathrm{radical}=\frac{Blankabsorbance-Sampleabsorbance}{Blankabsorbance}\times 100$$

#### 2.9.3. Hydroxyl radical (OH^⋅^) scavenging activity

With some modifications, the OH^⋅^ scavenging capacity of MPPE was determined according to the method described by Smirnoff and Cumbes (44). 1 mL of MPPE concentrations (50–400 μg mL^–1^) were mixed with 1 mL of FeSO_4_, saffron (360 μg mL^–1^), 1mL H_2_O_2_, and 0.5 mL of methanol-salicylic acid solution and given a standing time of 30 min at 37°C. The absorbance of the mixture was read at 520 nm against a blank. The reaction of plant extracts decreased the absorbance of bleached saffron, which specifies the decrease in hydroxyl radical scavenging capacity. % inhibition of OH^⋅^ was calculated using the following equation:

$$\%\mathrm{Inhibition}\mathrm{of}\mathrm{hydroxy}\mathrm{radical}=\frac{Blankabsorbance-Sampleabsorbance}{Blankabsorbance}\times 100$$

#### 2.9.4. Nitric oxide (NO^⋅^) scavenging activity

NO^⋅^ scavenging activity of MPPE was assessed by Griess reaction (45). 1 mL of MPPE concentrations were mixed with 2 mL of sodium nitroprusside, 0.5 mL of phosphate buffer saline, and incubated at 25°C for 3 h. After incubation, 1 mL of sulfanilic acid reagent (0.33% in 20% glacial acetic acid) was added, and the mix was kept stable for 5 min to complete diazotization. Then 1 mL of naphthyl ethylene diamine dihydrochloride was added, then incubated at 25°C for 30 min. The sample color was read at 540 nm absorbance against a blank sample.

$$\%\mathrm{Inhibition}\mathrm{of}\mathrm{nitric}\mathrm{oxide}\mathrm{radical}=\frac{Blankabsorbance-Sampleabsorbance}{Blankabsorbance}\times 100$$

### 2.10. Antidiabetic activity

#### 2.10.1. Inhibition of α-glucosidase enzyme

The MPPE inhibition ability of α-Glucosidase was assessed following by Bljajić et al. (46) method with slight modifications. 1 mL of MPPE concentrations mixed with α-glucosidase (0.2 U mL^–1^ in 0.1 M phosphate buffer, pH 6.9), then incubated for 10 min at 37°C. After incubation, 50 μL p-nitrophenyl-α-D-glucopyranoside solution (1mM in the same buffer) was added to the previous mix and incubated for 5 min to release p-nitrophenol. Finally, the absorbance was read at 405 nm. % Inhibition of α-glucosidase was calculated using the following equation:

$$\alpha -\mathrm{glucosidase}\mathrm{inhibition}\left(\%\right)=\left[\frac{A_{standard}-A_{sample}}{A_{standard}}\right]\times 100$$

#### 2.10.2. Inhibition of α-amylase enzyme

The inhibition ability of α-amylase was assessed by Apostolidis, Kwon (47). 0.5 mL of MPPE concentrations were added with standard (acarbose 1mg mL^–1^ in water) and 500 μL of the α-amylase enzyme (0.8 U mL^–1^ in 20 nM phosphate buffer, pH 6.8) of the porcine pancreas and incubated for 10 min at 25°C, followed by supplementation of 500 μL of soluble starch (0.5% solution in buffer) and standing for 10 min at 25°C. The solution was then added by 1 mL of 3.5-dinitro salicylic acid (96 mM), placed in boiling water for 5 min, and then cooled to room temperature. The plant mixture was diluted with 10 mL of distilled water, and its absorbance was read at 540 nm. The percentage of α-amylase inhibition was calculated using the following equation:

$$\alpha -\mathrm{amylase}\mathrm{inhibition}\left(\%\right)=\left[\frac{A_{standard}-A_{sample}}{A_{standard}}\right]\times 100$$

### 2.11. Cell culture

Caco-2 cells were cultivated in sterile RPMI-1640 medium supplemented with 100 μg mL^–1^ streptomycin, fetal bovine serum (FBS, 10%), and 100 U penicillin mL ^–1^, and incubated at 37°C in carbon dioxide (5 %). The culture medium was renewed daily and passed a few times until the cells achieved desired 80–90 % confluence (48).

### 2.12. Cytotoxicity evaluation by MTT assay

Caco-2 cells were tested for viability using MTT colorimetry to determine the effects of eight MPPE on the intestinal cells. The cell density of Caco-2 cells was measured with a hemocytometer. In the logarithmic growth phase, they were seeded onto 96-well plates in 3 × 10^4^ concentration and incubated for 24 h. Afterward, the medium was withdrawn, and cells were rinsed. Phosphate buffered saline (PBS) solution and then various MPPE was added to different cell-containing wells at varying concentrations (25, 50, 100, 200, and 400 μg mL^–1^) before being incubated for 24 h. The growth medium was again withdrawn, and the cells were washed with PBS solution. Then, 100 μL of MTT solution (0.5 mg mL^–1^) was added to each well and incubated for 4 h; 150 μL of DMSO solution was added and further incubated for 30 min. The absorbance of the control and treated cells was read at 570 nm using a microplate reader. The cell viability was evaluated in the following equation (48).

$$\mathrm{Cell}\mathrm{survival}\mathrm{rate}\left(\%\right)=\frac{\mathrm{OD}\mathrm{test}\mathrm{group}-\mathrm{OD}\mathrm{blank}\mathrm{group}}{\mathrm{OD}\mathrm{negative}\mathrm{group}-\mathrm{OD}\mathrm{blank}\mathrm{group}}\times 100$$

### 2.13. Antibacterial activity

The antibacterial activity of eight MPPEs was determined using the agar well diffusion method with some modifications (49, 50). Four species of pathogenic bacteria, particularly two gram-positive *Staphylococcus aureus*, *Bacillus cereus*, and two gram-negative *Escherichia coli* O157: H7 strains of *Salmonella enterica*, were used. Overnight bacterial cultures in nutrient broth were standardized using the McFarland method (51). 0.05 McFarland standard solution was prepared by mixing 500 μL of BaCl_2_ and 99.5 mL of H_2_SO_4_ to obtain a standard with an optical density comparable to a bacterial suspension with 1.5 × 10^8^ (CFU mL ^–1^) cell count. Each bacterial culture (200 μL) was spread on nutrient agar plates in duplicate. Wells were made with the help of a sterile hot cork borer (8 mm deep and approximately 2 cm apart) in these media plates, then 7 mm discs saturated with different concentrations of MPPE were placed in respected wells. Antimicrobial activity was sequentially noticed against all pathogens utilized in the study, along with the control, which was chloramphenicol antibiotic, after incubation at 37°C for 18–24 h. The antimicrobial activity of each plant extract against pathogenic bacteria was determined as the mean diameter of the inhibition zone (mm) around each well.

### 2.14. Statistical evaluation

All results in the study were expressed as means of triplicate data and statistically evaluated by Statistics software (Version 8.1, Florida) using a completely randomized design at 5% level of significance. Principal component analysis was applied to MPPE using Minitab software (Version 18.1, USA) to assess the difference between MPPE and identify characteristics related to their total polyphenols, total flavonoids, antioxidant activity, enzyme inhibition, and antimicrobial activity. Graphpad prism software (Version 8, USA) was used to identify the study’s 50% inhibition IC50 value of various parameters.

## 3. Results and discussion

### 3.1. Extraction yield of MPPE

The percentage extraction yield of plants with 80% methanol in the present study was 19–26% (Table 1). The maximum yield of polyphenols was obtained for the plant *C. sinensis* (25%), while the minimum extraction yield was obtained for the plant *M. longifolia* (19%). The results corresponded with the study of Safdar et al., who revealed that powdered mango peels produced a maximum extraction yield of 25.25% with 80% methanol (52). Polyphenol extraction from plants depends on the pretreatment processes (53), extraction method (54), temperature extraction (55), solvent type (56), and storage condition (55). It has been reported that the blanching process increased polyphenol content in the plant (17). Traditionally, maceration and thermal processes such as heating, boiling, and reflux have removed antioxidant compounds. Nevertheless, these affect the polyphenols’ stability and antioxidant capacity due to temperature effects and longer extraction times (57). Alternative technologies, such as ultrasound, have positive effects when applied for polyphenols extraction in kinnow (*Citrus reticulate* L.) peel (58).

### 3.2. Phytochemical qualitative analysis

The phytochemical analysis of the herbaceous and woody plants used in the study is summarized in Table 2, which revealed that the extract of *M. arvensis* contained all listed phytochemical constituents. Alkaloids, phenols, and flavonoids were ubiquitous in all plants’ extracts, while *M. longifolia* lacked steroids and *T. cordifolia* lacked glycosides. The results agree with previous studies; medicinal plants are rich sources of phytochemicals, alkaloids, phenols, flavonoids, carbohydrates, tannins, steroids, terpenoids, saponins, and glycosides (59–61). Medicinal plants are rich in secondary metabolites, a diverse group of chemicals, which include alkaloids, glycosides, amines, insecticides, steroids, flavonoids, and related metabolites, which have been extensively used in the drug and pharmaceutical industry. Many of the plant secondary metabolites are constitutive and exist in healthy plants in their biologically active forms. Still, others occur as inactive precursors and are activated in response to tissue damage or pathogen attack. The beneficial medicinal effects of plant materials typically result from the combinations of secondary products present in the plant, such as alkaloids, steroids, tannins, flavonoids, resins, fatty acids, etc. (62, 63).

### 3.3. Polyphenols content

#### 3.3.1. Total polyphenolic content (TPC)

**FIGURE 1:**
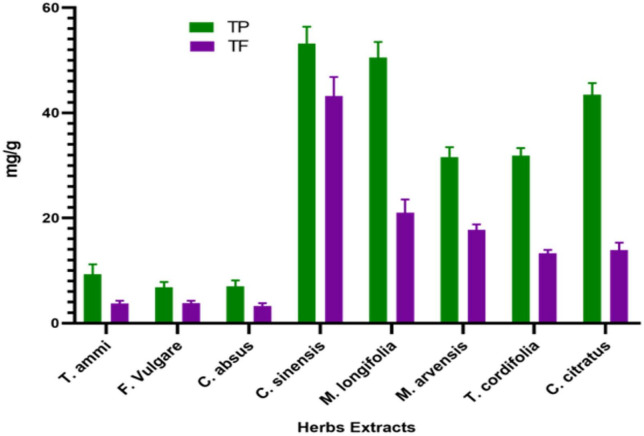
Total phenolic and flavonoid content (mean ± standard deviation) of dietary and medicinal plant extracts.

Plants contain phenolic compounds with antioxidant properties. Flavonoids are essential to these substances, possessing a wide range of chemical and biological activities, including radical scavenging properties (64). Therefore, the content of phenolic and flavonoid compounds of medicinal plant extracts was determined in the present study. The TPC of the dietary plants was found to be significantly different at (*p* < 0.05), as (Figure 1).

The *C. sinensis* has the highest TPC of 53.18 ± 3.18 mg GAE g^–1,^ followed by *M. longifolia* and *C. citratus* with 50.507 ± 2.96 and 43.433 ± 2.22 mg GAE g^–1^, respectively. The results follow the study of Turkmen et al., who reported that *C. sinensis* and *C. citratus* give high polyphenol yields (65). However, our study’s TPC of wild peppermint was markedly lower than the previously reported TPC value of 71.43 mg GAE g^–1^ (66). The lowest polyphenolic content concentration was 6.8267 1.02 mg GAE g^–1^ in the *F. vulgare* 6.8267 ± 1.02 mg GAE g^–1,^ as depicted in Figure 1.

Higher total polyphenolic content in tea and wild peppermint leaves is attributed to a phenolic hydroxyl group bond in the flavan-3-ol structure with a strong reducing ability (67). These phenolic compounds may improve foods’ shelf life and antimicrobial properties besides retention of color, taste, and flavor. They are the defense system to neutralize reactive oxygen species (ROS), preventing molecular damage and harmful effects on microorganisms and insects and thus increasing their survival ability (68).

#### 3.3.2. Total flavonoid content (TFA)

Flavonoids constitute an exceptional class of phenolic compounds with higher antioxidant activity than other phenolic acids. The carbon skeleton of diphenyl propane, which consists of two phenolic rings united with a heterocyclic carbon, is the reason for the strong functional properties of flavonoids (69).

In this colorimetric method, aluminum chloride reacts with adjacent keto or hydroxyl groups of flavones or flavonols to produce acid-stable complexes that quantify the content of flavonoid content of plants (70). The study’s total flavonoid content (TFC) of eight medicinal plants ranged from 3.29 ± 0.50 to 43.19 ± 3.63 mg QE g^–1^, as shown in Figure 1. All plants exhibited significant flavonoid content; however, tea leaves had the highest flavonoid content, 43 mg QE g^–1^, while the smallest flavonoid content, 3.29 mg QE g^–1^, was obtained in the *C. absus* seed extract. Our results resembled the study of (44), who also reported a high level of flavonoids in tea leaves. Moreover, slight variations in results than previous studies may be due to the difference in sample collection, extraction method, or environmental conditions. The high flavonoid content in tea extract is due to many caffeoyl derivatives in tea leaves, such as caffeic acid, chlorogenic acid, and caffeoylquinic acid, which are significantly responsible for their antioxidant capacity (71). The total flavonoid contents of plants do not conflict with their total phenolic contents, so they can possess a high amount of both functional compounds simultaneously.

The number of flavonoids in *M. longifolia* was marginally higher than the number of flavonoids (21.0 ± 2.52 mg CE g^–1^) in *M. arvensis* (17.75 ± 1.03 mg CE g^–1^). Hajlaoui et al. reported markedly higher flavonoid contents in their study for *M. pulegium* and *M. longifolia* 63.93 and 33.83 mg CE g^–1^, respectively; this probably depends on their habitats (72). Flavonoids help plants produce metabolizing enzymes such as quinone reductase, glutathione-*S*-transferase, and uridine 5-diphospho-glucuronyl transferase that detoxify carcinogens and help in their elimination from the body, which is similar to the mechanism of chemotherapy. Due to their antioxidant, anti-inflammatory, antimutagenic, anticarcinogenic, and enzyme-modulatory functions, flavonoids are essential components in various nutraceutical, pharmaceutical, and cosmetic applications in today’s world (73).

#### 3.3.3. FTIR analysis of active groups in MPPE

**FIGURE 2:**
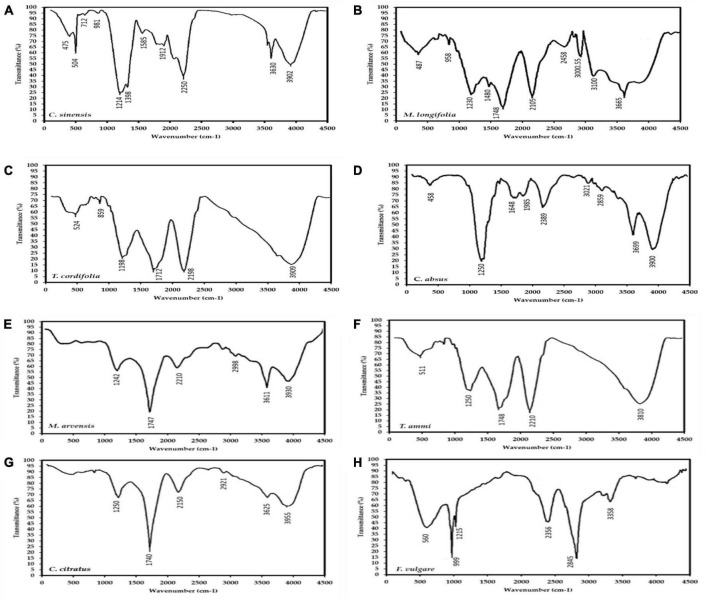
FTIR spectrum of eight medicinal plants extracts (MPPE), i.e., **(A)** *Camellia sinensis*, **(B)** *Mentha longifolia*, **(C)** *Tinospora cordifolia*, **(D)** *Cassia absus*, **(E)** *Mentha arvensis*, **(F)** *Trachyspermum ammi*, **(G)** *Cymbopogon citratus*, **(H)** *Foeniculum vulgare*, showing the active groups that may be related to polyphenols.

The active groups in the MPPE were characterized by FTIR analysis to distinguish the nature of phytogenic compounds in MPPE (monomer or polymer). Figure 2 depicts eight plants’ FTIR absorption spectra of MPPE, i.e., *Mentha longifolia, M. arvensis, Tinospora cordifolia, Cymbopogon citratus, Foeniculum vulgare, Cassia absus, Camellia sinensis*, and *Trachyspermum ammi*. *C. sinensis* and *M. arvensis*. Absorbance bands detected in the range 500–4,000 cm^–1^ in Figures 2A–H are about thirty-two bands seen in the eight plants; 3,955, 3,930, 3,810, 3,665, 3,625, 3,611, 3,358, 3,100, 3,000, 2,998, 2,921, 2,458, 2,845, 2,356, 2,210, 2,150, 1,985, 1,748, 1,747, 1,648, 1,585, 1,480, 1,398, 1,242, 1,215, 1,250, 999, 859, 712, 560, 511, 504, 475, 458 cm^–1^. These bands correlated to the stretching vibrations of –C–O–C–, ether bonds, –C–O– terminal methyl, –C–C– groups, aromatic rings, alkyne bonds, and N–H, primary and secondary amines, and O–H groups, respectively.

These bands represent the vibrational stretching bands responsible for compounds such as terpenoids and flavonoids. The 1,640–1,550 cm^–1^ absorption peak corresponding to proteins suggests that proteins interact with polyphenol monomers. Additionally, Absorption peaks observed between 3,950 and 3,200 cm^–1^ imply alcohols (O–H bands) presumably originating from proteins and carbohydrates present in the sample, which bind with polyphenols and are responsible for stability and increasing the activity of produced polymers. These active groups prove that MPPE contains polyphenolic polymers conjugated with carbohydrates and proteins that enhance their bio-availability and bio-activity. Based on Figures 2A–C, *C. sinensis* polyphenolic extract exhibited 11 bands from 3,902 to 475 cm^–1,^ followed by *M. longifolia* extract with ten peaks from 3,665 to 487 cm^–1^, then *C. absus* phenolic extract with nine peaks (3,900–458 cm^–1^).

Muruganantham et al. (74) studied the FTIR spectrum of medicinal plants’ different parts, such as leaf, stem, and root. The false daisy (*Eclipta alba* and *E. prostrate*) extracts revealed the presence of bioactive groups such as COO, NH_2_, NH, sulfur derivatives, carbohydrates, NO, chlorates, and carbohydrates. These active groups are responsible for the various medicinal properties. At the same time, the active groups of methanolic and aqueous extracts of *Bauhinia racemosa* leaf extracts revealed the presence of proteins, oils, lipids, phenolic compounds, flavonoids, saponins, tannins, and carbohydrates in FTIR spectra (75). Furthermore, Ragavendran et al. (76) detected COO, NH, NH-CO, polysaccharides, sulfur derivatives, halogen, and hydrocarbons responsible for the various therapeutic actions of *Aerva lanata*. Similarity, the same active groups were detected in black creeper (*Ichnocarpus frutescens*) ethanolic extract (77).

Also, Pednekar and Raman (78) found that the wild grape (*Ampelocissus latifolia*) methanolic leaf extract had transition metal carbonyl compounds and aliphatic fluoro compounds. The active compounds in *Senna auriculata, Solanum torvum, Phyllanthus maderaspatensis, and Phyllanthus amarus* leaves methanolic extracts act as natural antibiotics. The detected active groups in these plants indicate the existence of amino acids, amides, lipids, carbohydrates, glycogen, cellulose, starch, carotenoids, calotropin, and calotropogenin phosphates. The common active group in all extracts was OH, which can create hydrogen bonds and has the potential to act as an antibacterial agent at low IC50 (79).

It is well known that polymers such as proteins and carbohydrates can stabilize and enhance the bioavailability of medicinal medicines. Consequently, they can also be employed to improve the stability of polyphenols by polymerization or conjugation with polymers. The synthesized quercetin polymers exhibited antioxidant and antibacterial properties against three prevalent bacteria: *B. subtilis, E. coli*, and *S. aureus*. Sahiner re-established antioxidant and antibacterial activities by polymerizing rutin acid with the same approach (80, 81). In addition, Sahiner et al. (81) revealed that tannic polymers were antioxidant and anticancer properties against A549 malignant cells compared to cisplatin. In contrast, rutin and tannic acid polymers exhibited excellent drug-release properties.

Quercetin and kaempferol polymers are catalyzed with enzymes with enhanced antioxidants compared to their respective monomers, while oligomeric catechin from HRP exhibits antibacterial activities (82). The polymerization of tannic acid with laccase resulted in the formation of gallic dimers, gallic, partly gallic-glucose ester, and glucose. 68 Several phenolic acids have also been combined with chitosan enzymatically. Balasubramaniam, Murugan (83) used the polymerization of ferulic with chitosan by laccase to improve its antioxidant and antibacterial activity –comparable to the native chitosan activity. Božič, Štrancar (84) studied the effect of pH on phenolic-chitosan polymer activity. They found that pH 4.5 exhibited the most potent antioxidant activity and increased antibacterial activity against *Listeria monocytogenes* and *Escherichia coli*, compared to native chitosan. As we know that MPPE contains active groups related to polyphenols, proteins and carbohydrates detected by FTIR, therefore the UHPLC analysis was conducted to detect the phenolic compounds in MPPE in the following section:-

#### 3.3.4. UHPLC- phenolic profile in MPPE, their uses, and safety

HPLC detected Twenty-five phenolic compounds in eight medicinal plant extracts (Table 3), categorized under three groups: phenolic acids, flavonoids, and alkaloids. Following FTIR results, four medicinal plants in descending order have the most detected phenolic compounds, i.e., *C. sinensis* > *M. longifolia* > C. *absus* > *T. cordifolia*. In the HPLC profile of *C. sinensis*, 22 compounds were found. The main compound was 3-Feroylquinic acid (1,302 mg L^–1^), also found in *M. longifolia* and *C. absus*. *C. sinensis* has a higher phenolic content, i.e., vanillic acid, ferulic acid, chlorogenic acid, p-coumaric acid, gallic acid, rosmarinic acid, catechin, luteolin, 7-*O*-neohesperideside, quercetin 3,7-*O*-diglucoside, Hesperidin, Rutin, quercetin, and caffeine in the range of (560–780 mg L^–1^). At the same time, other compounds are of medium content (99–312 mg L^–1^). The phenolics in *C. sinensis* were 20–116% more than those in *M. longifolia* and *C. absus*. On the other hand, *C. absus is* characterized by sinapic acid (450.69 mg/L). At the same time, Carlinoside is the precious compound in *C. citratus* (525.98 mg L^–1^). *Additionally*, genkwanin 5-*O*-glucoside is the main compound in *M. longifolia*. *F. vulgare* is rich in quercitron and quercetin dihydrate (803–900 mg L^–1^). Also, *T. cordifolia* is rich in alkaloids (palmatine, caffeine, and berberine). *T. ammi* had a lower content of detected polyphenols than other medicinal plants.

**TABLE 3:** Phenolic compound profile of eight medicinal plants.

Polyphenolic compounds	Medicinal plants (mg/L)							
	*C. citratus*	*T. cordifolia*	*M. arvensis*	*M. longifolia*	*C. sinensis*	*C. absus*	*F. vulgare*	*T. ammi*
**Phenolic acids**								
Vanillic acid	599.98 ± 0.7	108.46 ± 0.7	106.67 ± 1.5	561.16 ± 0.8	690.13 ± 0.9	318.46 ± 0.1	118.1 ± 0.2	105.36 ± 0.5
Ferulic acid	224.16 ± 0.8	110.42 ± 0.5	108.63 ± 1.3	563.12 ± 0.6	692.09 ± 1.5	320.42 ± 0.5	120.06 ± 0.5	107.32 ± 0.8
Chlorogenic acid	440.3 ± 0.6	202.13 ± 0.5	200.34 ± 0.3	654.83 ± 0.9	783.8 ± 0.4	412.13 ± 0.8	211.77 ± 0.9	199.03 ± 0.2
p-coumaric acid	801.25 ± 0.5	189.91 ± 0.9	188.12 ± 0.8	642.61 ± 0.3	771.58 ± 0.3	399.91 ± 0.5	199.55 ± 1.1	186.81 ± 0.3
Gallic acid	78.57 ± 0.8	182.07 ± 1.3	478.91 ± 0.9	512.33 ± 0.1	591.92 ± 0.9	79.72 ± 0.8	87.1 ± 0.2	181.09 ± 1.1
Rosmarinic acid	212.29 ± 0.2	98.55 ± 0.6	96.76 ± 0.2	551.25 ± 0.5	680.22 ± 0.8	308.55 ± 0.9	108.19 ± 0.5	95.45 ± 0.6
Sinapic acid	ND	23.45 ± 0.8	ND	84.25 ± 0.3	99.45 ± 0.2	450.69 ± 0.4	ND	ND
3-Feroylquinic acid	382.33 ± 0.9	211.56 ± 0.1	195.88 ± 0.5	1,105.69 ± 1.5	1,302.33 ± 0.3	897.33 ± 1.2	456.3 ± 0.1	122.65 ± 1.2
Neochlorogenic acid	216.4 ± 0.1	102.66 ± 0.7	100.87 ± 0.8	555.36 ± 0.2	684.33 ± 0.2	312.66 ± 0.4	112.3 ± 0.2	99.56 ± 0.3
**Flavonoids**								
Carlinoside	525.89 ± 1.1	ND	ND	ND	102.33 ± 0.8	ND	ND	ND
Catechin	510.21 ± 1.1	18.69 ± 0.8	16.9 ± 0.2	471.39 ± 0.2	600.36 ± 0.3	228.69 ± 0.2	28.33 ± 0.9	15.59 ± 0.8
Luteolin 7-*O*-neohesperidoside	112.35 ± 0.2	215.85 ± 0.8	512.69 ± 0.1	546.11 ± 0.2	625.7 ± 1.1	113.5 ± 0.2	120.88 ± 1.3	214.87 ± 0.4
Quercetin 3,7-*O*-diglucoside	195.05 ± 0.1	81.31 ± 0.7	79.52 ± 0.9	534.01 ± 0.3	662.98 ± 1.8	291.31 ± 0.8	90.95 ± 0.2	78.21 ± 0.1
Luteolin 5-*O*-glucoside	120.65 ± 0.2	12.30 ± 1.1	90.15 ± 0.2	240.35 ± 0.5	312.55 ± 0.9	201.23 ± 0.8	99.11 ± 0.1	87.39 ± 0.3
Genkwanin 5-*O*-glucoside	ND	ND	ND	550.39 ± 1.1	ND	ND	ND	ND
Hesperidin	161.04 ± 1.5	47.3 ± 0.1	45.51 ± 0.9	500.00 ± 0.2	628.97 ± 0.3	257.3 ± 0.2	56.94 ± 0.7	44.2 ± 0.7
Diosmin	ND	ND	195.23 ± 0.4	85.33 ± 0.4	105.11 ± 1.9	ND	ND	ND
Didymin	ND	ND	250.11 ± 0.5	ND	ND	ND	ND	ND
Rutin	237.03 ± 0.9	123.29 ± 0.0	121.5 ± 0.1	575.99 ± 0.3	704.96 ± 0.2	333.29 ± 0.3	132.93 ± 0.1	120.19 ± 0.2
Quercetin dihydrate	95.27 ± 0.5	ND	ND	434.23 ± 0.1	563.2 ± 0.2	191.53 ± 0.1	803.04 ± 1.0	ND
Kaempferol	ND	55.35 ± 0.3	ND	125.56 ± 0.9	210.65 ± 0.8	100.36 ± 0.4	57.36 ± 0.2	100.87 ± 0.9
Quercetin	207.7 ± 0.2	93.96 ± 0.5	92.17 ± 0.8	546.66 ± 0.5	675.63 ± 0.3	303.96 ± 0.6	900.6 ± 0.8	90.86 ± 0.1
**Alkaloids**								
Palmatine	ND	450.22 ± 0.2	ND	ND	ND	ND	ND	ND
Caffaine	474.3 ± 1.8	11.23 ± 2.1	50.66 ± 0.1	435.48 ± 0.8	564.45 ± 0.2	192.78 ± 0.7	21.87 ± 0.8	ND
Berberine	ND	862.22 ± 0.9	ND	ND	99.32 ± 0.7	ND	ND	ND

*n* = 3, Data are presented as mean ± SD. ND, non detected.

About two-thirds of the phenolics in plant-based foods are flavonoids, which are very important to human health. For instance, caffeine releases fatty acids from stored body fat and inhibits fat absorption, resulting in weight loss. Quercetin’s antioxidant and anti-inflammatory properties may help decrease edema, eliminate cancer cells, regulate blood sugar, and prevent heart disease.

Nevertheless, phenolic acids represent one-third of polyphenols. Due to their antioxidant qualities and predicted health benefits, the scientific community’s interest in these simple phenolic acids continues to increase (23). Phenolic acids spread in many foods, i.e., fruits, cereals, vegetables, tea/coffee, spices, etc.; humans need about 25 mg daily (24).

Phenolic acids, which have a variety of biological uses, are the primary polyphenols generated by plants and serve as the progenitor of bioactive compounds often employed in the pharmaceutical, cosmetic, and food sectors. Phenolic acids are essential for absorbing nutrients, building parts, enzyme activity, protein structure, photosynthesis, and plant communication. The primary benefit of employing phenolic acids is their capacity to be metabolized by natural bacteria; hence, they provide an essential alternative to artificial chemicals that are equally damaging to the environment. While neochlorogenic acid acts as an antioxidant that scavenges potentially harmful free radicals and has been proven to inhibit bone resorption and promote bone formation (85), it also inhibits bone resorption. While chlorogenic acid decreases food cravings, reduces daily calorie intake, and induces body fat reduction through heat production (86), similar to statins used to treat cholesterol and triglyceride issues.

Abundant evidence confirms that a diet rich in vegetables and fruits improves health since they primarily function as antioxidants and prevent oxidative damage-induced illnesses such as stroke, coronary heart disease, and cancer (87–89). The average daily consumption of phenolic acids is 211 mg/day for hydroxy-cinnamic acid and 11 mg/day for hydroxybenzoic acid. At the same time, the population tested exhibited a range of 6 to 987 mg of phenolic acids (90). Exploring the probable significance of phenolic acids in plant life is only one of several research projects; their involvement in food quality is also a major field of study. They have improved foods’ sensorial, antioxidant, and nutritional properties (91).

Besides their roles as food preservatives, the phenolic acids affect fruit ripening and inhibit enzymatic browning (23, 92). Caffeic and ferulic acids (free/esterified) are the most prevalent phenolic acids in most fruits and cereal grains. Also, alkaloids are valuable therapeutic compounds that may be used to treat various disorders, such as malaria, diabetes, cancer, and cardiac dysfunction; for instance, palmatine and berberine play a crucial role in osteoclast apoptosis via the NOS system in osteoclasts (93). It has been claimed that palmatine possesses sedative and antioxidant properties. Palmatine enhances memory in mice by reducing brain acetylcholinesterase activity via the GABA-benzodiazepine pathway and its antioxidant properties (94). Inhibitors of beta-site amyloid precursor protein-cleaving enzyme 1 (BACE 1), acetyl- and butyrylcholinesterases (AChE and BChE) have also been identified (94).

Herbal remedies are frequently utilized as alternatives to pharmaceuticals. According to traditional Indian literature, *Tinospora cordifolia* (Willd.) Miers (TC), a member of the Menispermaceae family, is a well-known traditional herb used to cure diabetes mellitus and various other diseases. Herbal anti-diabetic medications containing TC are commercially available. TC has been found as a potent CYP2C9 inhibitor, thereby increasing the likelihood of herb-drug interactions (HDI) with drugs metabolized via the CYP pathway. *In vivo* pharmacokinetic research (95). examined the pharmaco-kinetic HDI of TC extract with Gliclazide (GL) following oral co-administration in Wistar rats. In addition, Mentha arvensis and Mentha longifolia have substantial pharmacological uses as antioxidants, anti-fertility agents, anti-hepatoprotective agents, and antibacterial, antifungal, antimicrobial, anti-inflammatory, and anti-viral agents, among others. Due to phenolic and flavorful essential components, the wild mint essential oil has demonstrated remarkable biological applications (96).

The leaf of *Cymbopogon citratus* is also used orally with mate tea in Argentina for sore throat, empacho, and as an emetic (97). Gianeti, Mercurio (98) found that green tea-based cosmetics helped keep the skin moist and positively affected the skin’s microrelief. Traditional medicine also uses *Cassia absus* to treat bronchitis, asthma, cough, leucoderma, renal and hepatic illnesses, constipation, tumors, venereal ulcers, headaches, hemorrhoids, and wound healing. Preliminary research *in vitro* and *in vivo* has shown crucial scientific evidence for its use (30).

### 3.4. Antioxidant activity of MPPE

#### 3.4.1. DPPH free radical scavenging activity

The antioxidant activity of MPPE was also examined as a free radical scavenging response using a DPPH assay. Free radicals play a significant role in chronic diseases such as cancer, diabetes, cardiovascular diseases, arthritis, and neural diseases (99). Antioxidants react with the DPPH substance, reducing its oxidative properties, proportional to the amount of hydroxyl (OH-) ions prevailing in the reaction mixture. Currently, the research on medicinal plants is more focused on discovering free radical-scavenging substances that translate into their antioxidative, antiradiation, anti-lipid oxidation, and immune modulation properties.

**FIGURE 3:**
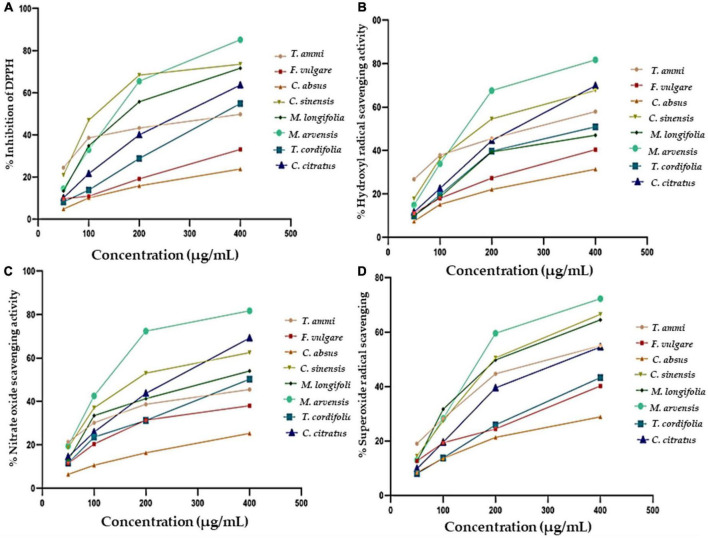
The antioxidant activity of eight different methanolic plant extracts as measured by different methods: **(A)** DPPH radical scavenging activity. **(B)** Hydroxyl radical scavenging activity. **(C)** Nitric Oxide Scavenging activity. **(D)** Superoxide radical scavenging activity.

Eight medicinal plants investigated in the study demonstrated considerable antioxidant abilities (Figure 3A). The scavenging ability of MPPE against DPPH* was found to be 4.8 ± 0.92 to 85.21 ± 3.67% for different concentrations varying between 50 and 400 μg. The highest inhibition activity was represented by *M. arvensis* (20.97 ± 1.39 to 73.57 ± 2.29%, IC_50_ value 119.9 ± 2.9 μg mL^–1^), and *C. sinensis* (13.34 ± 1.68 to 71.68 ± 2.89%, IC_50_ value 130.3 ± 2.7 μg mL^–1^) at various concentrations. The lowest percentage of inhibition was found in *F. vulgare* and *C. absus*, which fluctuated between 4.8 ± 0.96 and 23.8 ± 1.8% with the IC_50_ value (194 ± 2.7 μg mL^–1^).

An earlier study by Zakaria et al. reported that the antioxidant activity of *M. arvensis* extract was 66.37% with a 100% methanol concentration (100). This variation in the antioxidant activity of *M. arvensis* may be due to the difference in the analytical method, which was also described by Safdar et al., that antioxidant activity decreases by increasing methanol purity (52). *M. longifolia, C. citratus, T. cordifolia*, and *T. ammi* exhibited significant antioxidant activity, which tallied the findings of earlier researchers with some differences (101). The present investigation also affirmed that the scavenging activity is directly proportional to the concentration of plant extracts. All plant samples showed maximum scavenging activity at a concentration of 400 μg, which progressively declined with decreasing concentration until the slightest scavenging activity was observed at 50 μg. Ashraf et al. documented a similar relationship in their experiment (70).

Phenols and flavonoids are well-known antioxidant compounds. In flavonoids, the presence of an O-dihydroxy structure on the B-ring that contains 2, 3 double bonds conjugated with a 4-oxo function on the C-ring; the 3-hydroxyl ions on the C-ring at positions C3 and C5 bonded with 4-production on the A and C-ring are responsible for a high free radical scavenging potential(102). However, in phenolic compounds acids such as 2,3-hydroxybenzoic acid, caftaric acid, gallic acid, and caffeic acid, a 3-hydroxyl structure intensified the antioxidant properties (1, 102).

#### 3.4.2. Hydroxyl radical scavenging activity

Hydrogen radicals are considered among the most active and dangerous free radicals for human health among reactive oxygen species, causing cell injury at the site of inflammation in oxidative stress-induced diseases (103). The antioxidant system in the human body protects cells from free radicals once they are produced. Antioxidants are produced in the body by consuming a diet rich in phytochemicals which give rise to transferase, lipase, and protease enzymes involved in DNA repair. Moreover, catalase, Se-glutathione peroxidase, and superoxide dismutase are also produced, which play a major role in enzymatic defense, hydrolyzing superoxide, lipid peroxide, and hydrogen peroxide, thus preventing toxic radicals. Hydroxyl radical (OH) is the most powerful oxidant from exposure to the Fenton reagent (104).

All plants showed significant dose-dependent hydroxyl radical activity in the present study, as shown in Figure 3B. *M. arvensis* and *C. citratus* showed the highest hydroxyl scavenging activity compared to other vegetal extracts with IC_50_ values of 133.3 ± 3.4 and 174.9 ± 3.1, respectively, at the concentration of 400 μg mL^–1^ (Table 4), followed by *C. sinensis*, *T. ammi, T. cordifolia, M. longifolia. C. absus* and *F. vulgare* showed lower hydroxyl radical scavenging activity at all extract concentrations. The results are consistent with the previous literature reporting that the ethanolic extract of *M. arvensis* had good hydroxyl scavenging activity (70%) with a low IC_50_ value of 52 μg mL^–1^ (105). The variation in the result might be due to the plant sample, testing conditions, and the nature of the solvent used to prepare the extract. Furthermore, it is also documented that *C. citratus* (106) and *C. sinensis* (107) have potential hydroxyl radical activity due to the number of hydroxyl groups presents in the B ring of the flavonoid content of plant extracts.

**TABLE 4:** IC_50_ (mean ± standard deviation) value of different plants extracts against antioxidant, α-amylase inhibition, α- glucosidase inhibition, and Caco-2 cell line.

Plants extracts	IC_50_ (μg/mL)						
	Caco-2 cell	DPPH	NO	OH	O_2_^–^	α- glucosidase	α-amylase
*Trachyspermun ammi*	116 ± 4.4^b^	106 ± 1.9^a^	131 ± 2.1c	159 ± 1.7^e^	145 ± 2.6^d^	110 ± 1.9^b^	128 ± 1.1^c^
*Foeniculum vulgare*	121 ± 2.5^b^	212.7 ± 6.1^h^	120 ± 1.8^b^	184 ± 1.4^f^	193 ± 6.4^g^	125 ± 1.1^c^	117 ± 2.3^b^
*Cassia absus*	110 ± 1.0^a^	194 ± 2.7^g^	177 ± 5.9^f^	162 ± 2.2^e^	156 ± 3.2^e^	107 ± 1.7^a^	114 ± 1.5^b^
*Camellia sinensis*	189 ± 2.2^f^	100 ± 1.1^a^	117 ± 1.3^a^	97 ± 1.1^a^	148 ± 2.7^d^	122 ± 1.2^c^	113 ± 1.2^b^
*Mentha longifolia*	104 ± 4.6^a^	130.3 ± 2.7^c^	118 ± 1.4^b^	138 ± 1.4^c^	131 ± 2.1^c^	119 ± 1.5^b^	124 ± 2.8^c^
*Mentha arvensis*	111 ± 7.2^b^	143.4 ± 3.1^d^	118 ± 1.4^b^	130 ± 1.2^c^	136 ± 2.3^c^	146 ± 2.7^d^	109 ± 7.1^a^
*Tinospora cordifolia*	143 ± 2.0^d^	205 ± 5.7^g^	167 ± 3.1^e^	147 ± 2.1^d^	188 ± 6.2^f^	123 ± 6.2^c^	114 ± 2.5^b^
*Cymbopogon citratus*	128 ± 2.6^c^	195 ± 1.3^g^	189 ± 8.2^f^	182 ± 3.7^f^	156 ± 3.7^e^	124 ± 2.3^c^	107 ± 3.9^a^

*n* = 3, Data are presented as mean ± SD. Lowercase letters in the same column indicate significant differences at *p* < 0.05.

#### 3.4.3. Nitric oxide scavenging activity

The nitric oxide radical is the most toxic among all free radicals. It induces an inflammatory response, and the severity of damage increases with the presence of oxygen to produce peroxynitrite, which further harms living cells and their constituents. The generation of nitric oxide, followed by the combination of sodium nitroprusside and oxygen, forms nitrite. Plant extracts affect nitrite production by directly bonding with oxygen or reacting with NO. The NO scavenging activity of different plant extracts is shown in Figure 3C, indicating that the NO scavenging activity is directly proportional to the concentration of plant extracts. *M. arvensis* showed maximum NO scavenging activity (81.71 ± 1.1%) at all concentrations compared to the standard with IC_50_ values of 132.8 ± 3 and 118.3 ± 3.5 μg mL^–1^, respectively (Table 4). *C. citratus* and *C. sinensis* showed more than 50% NO scavenging activity. *M. longifolia, T. cordifolia, T.ammi*, and *F. vulgare* showed an average NO scavenging activity, while *C absus* showed the lowest NO scavenging activity with an IC_50_ value of 177.3 ± 2.8 μg mL^–1^.

Earlier literature showed that *M. arvensis* methanolic extracts have moderate NO scavenging activity at 71.28% at 250 μg mL^–1^ concentration due to the chelating ability of phenolic compounds (108). Similarly, previous studies reported bioactive compounds in *C. citratus*, *C. sinensis*, *M. longifolia*, *T. cordifolia*, *F. vulgare*, and *T.ammi* significantly inhibited *in vitro* NO generation that could mitigate harmful effects of inflammatory conditions (108–112). NO scavenging activity of plants originates from tannins, phenols, flavonoids, and alkaloid compounds in plants, particularly catechins in *C. sinensis* (113). This study implies that indigenous plants can be influential in preventing diseases triggered by nitric oxide.

#### **3**.4.4. Superoxide radical scavenging activity

The superoxide radical is formed in living organisms during aerobic respiration, which causes the generation of hydrogen peroxide. The high oxidizing activity of H_2_O_2_ badly affects the cells and damages their lipid, protein, and nucleic acid components. Their Antioxidant activity estimates the therapeutic activity of plant extracts. All plant extracts (Figure 3D) demonstrated considerable superoxide radical scavenging activity, which ranged from 28.83 ± 0.7% to 90.15 ± 1.8% at 400 μg mL^–1^ in a dose-dependent curve. *M. arvensis* and *C. sinensis* exhibited the highest inhibition of superoxide radicals with the IC_50_ value 144.1 ± 3.3 and 148 ± 2.7 μg mL^–1^, respectively. Extracts of *M. longifolia*, *T. ammi*, and *C. citratus* showed moderate superoxide scavenging activity. While *F. vulgare, T. cordifolia*, and *C. absus* showed the lowest percentage of superoxide inhibition with an IC_50_ value of 188.9 ± 3.3 and 156.1 ± 2.8 μg mL^–1^ (Table 4).

Our results follow Kumar et al., who reported that *T. cordifolia* has an average superoxide radical scavenging inhibition property comparable to the standard (114). The superoxide radical activity of the *C. absus* extract has not been reported earlier; however, methanolic extracts of *C. absus* showed lower DPPH inhibition percentage in the research of Ahmad et al. (30). Other research also corresponds to the present study’s findings that *M. arvensis* (115) and *C. sinensis* (116) have more vigorous superoxide radical scavenging activity than the standard. Previous investigations have also reported phenols, flavonoids, glycosides, phytochemicals (isoorientin, orientin, and isoscoperin), and sugar moieties at positions C-6, C-7, and C-8 as powerful sources of superoxide radicals in plants (117). Similarly, phytochemicals, including alkaloids, flavonoids, methoxy residue, disulfide bond, and hydroxyl groups in *M. arvensis* and *M. longifolia* and quercetin, epicatechin gallate, gallic acid, and epigallocatechin gallate in *C. sinensis* attributes to its higher superoxide anion scavenging potential (118).

### 3.5. Enzyme inhibition of MPPE

#### 3.5.1. α-glucosidase inhibition

**FIGURE 4:**
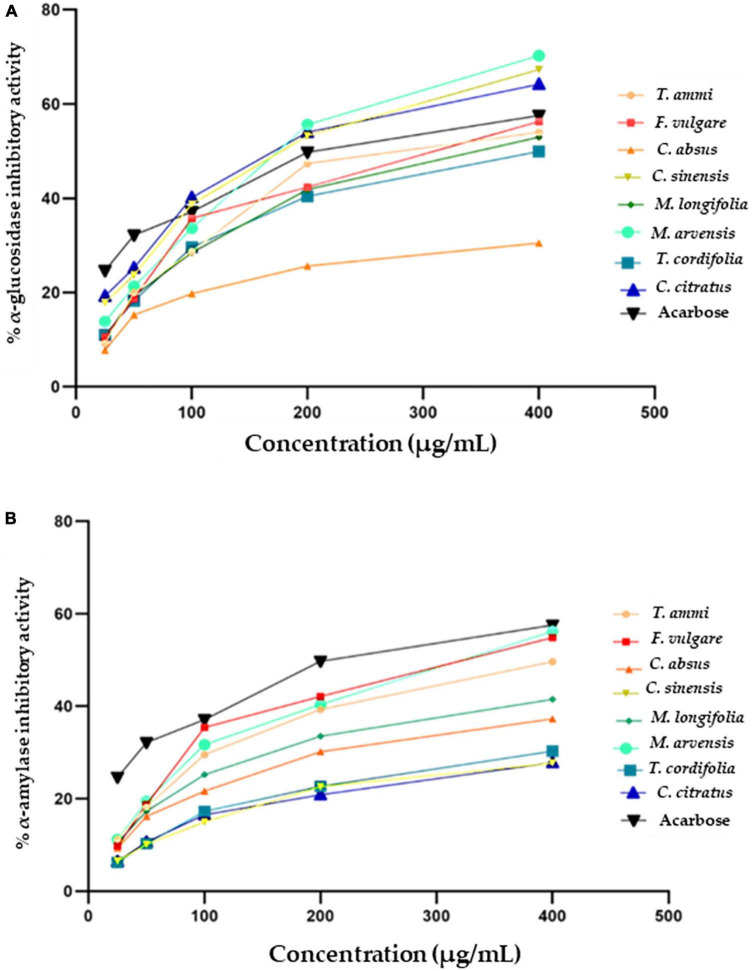
Antidiabetic activity of selected plant extracts and standard (Acarbose) **(A)** α- glucosidase inhibitory (%) activity **(B)** α-amylase inhibitory (%) activity.

Plant extracts can act as antidiabetic agents by their influence on enzymes crucial in carbohydrate digestion or by restricting the hydrolysis of carbohydrates to glucose and other simple sugar compounds. α-glucosidase enzymes convert carbohydrates to glucose in the small intestine after partial breakdown by an α-amylase enzyme from saliva and pancreatic juice. Plant substances are increasingly used to develop natural-origin medicines for curing diabetes, hyperglycemia, and obesity by inhibiting α-glucosidase enzymes. The α-glucosidase inhibition activity of eight MPPEs is demonstrated in Figure 4A. *M. arvensis*, *C. sinensis*, and *C. citratus* had the strongest inhibitory activity, which was even greater than the control Acarbose at 400 and 200 μg mL ^–1^ with the IC_50_ value 125.7 ± 2.4 and 124 ± 2.3 μg mL^–1^ (Table 4). *F. vulgare, T.ammi*, and *M. longifolia* were more than 50% inhibitive to α-glucosidase at (400 μg mL ^–1^) concentration. At the same time, *T. cordifolia* and *C. absus* recorded minor α-glucosidase inhibition activity at all concentrations used in the research.

The current study showed weaker inhibitory activity of methanolic extracts of *C. absus* than a previous contrasting study which reported that hydroalcoholic extracts of *C. absus* had potent inhibitory activity against α-glucosidase (119). However, The results of the present investigation are per the majority of earlier research, which reported that *C. citratus* (115), *M. arvensis* (120), *F. vulgare* (121) had strong α-glucosidase inhibition activity due to their high phytochemical contents, such as alkaloids, flavonoids, and saponins that bind other non-competitive enzymes (119).

*In vivo*, the antidiabetic and antihyperglycemic activity of streptozotocin in diabetic rats was caused by C-glycosyl flavones, *iso*-orientin, and chlorogenic acid naturally occurring in lemongrass extracts (117). Whereas the strong enzyme inhibition activity of *F. vulgare* extracts may be attributed to the presence of caffeic acid. An in vitro study showed that caffeic acid derivatives (122) and 3,4-di-*O*-caffeyolquinic acid (123) could inhibit the α-glucosidase activity and angiotensin-converting enzymes, which can reduce the risk of type-2 diabetes mellitus. The main mechanisms of caffeic acid, which regulates blood glucose levels, are stimulating insulin production, triggering glucose uptake by adipocytes in the liver, and reducing the glucose level in the bloodstream. In general, phenolic compounds can alter carbohydrate metabolism by their binding affinity towards hydrolyzing enzymes (121). Present study confirms the antidiabetic activity of selected plant extracts which indicates their suitability for use in therapeutic medicine.

#### 3.5.2. α-amylase inhibition

An effective strategy is required to tackle or manage diabetes mellitus by controlling or altering the activity of α-amylase, which prevents or delays the digestion of carbohydrates after their ingestion, eventually modulating the circulatory glucose level (124). Figure 3B depicts the antidiabetic potential of medicinal plant extracts as measured by their α-amylase inhibitory activity along with IC_50_ values (Figure 4B and Table 4). The percentage of inhibition of different plant extracts at a concentration of 400 μg mL ^–1^ against α-amylase is expressed in the following order *M. arvensis* > *F. vulgare* > *T.ammi* > *M. longifolia* > *C. absus* > *T. cordifolia* > *C. citratus* > *C. sinensis*. All vegetal extracts impaired α-amylase activity to appreciable levels. The extracts of *M. arvensis*, *F. vulgare*, and *T.ammi* showed the strongest inhibitory activity of α-amylase (IC_50_ 109 ± 7.1, 117 ± 6.3, and 128 ± 1.1 μg mL ^–1^) as compared to the extracts of other plants, while *C. sinensis* had the weakest inhibitory rate (27.69 ± 1%) with an IC_50_ value of 113 ± 1.2 μg mL ^–1^.

Similar results were reported by Wang et al., that 75% of ethanolic extracts of *C. sinensis* showed mild inhibition response against the α-amylase activity (125). Furthermore, the remarkable α-amylase inhibition activity of *M. arvensis*, *F. vulgare*, and some other Bangladeshi and Indian medicinal plants was also reported in some other studies (120, 121, 124). Another research highlighted that the α-amylase inhibition activity of medicinal plants could be associated with their phenolic compounds, which largely reduces the risk of developing type-2 diabetes among people (126, 127). Therefore, it can be inferred that the dietary plants utilized in the study can be used as medicine or functional food for treating or preventing diabetes mellitus along with healthy lifestyle changes.

Cell viability tested at maximum vegetal extract concentration (400 μg mL ^–1^) showed the least amount of living Caco-2 cells in the reaction mixture of *T. ammi, C. sinensis, C. citratus*, and *M. arvensis* 15.7 ± 0.4, 39.54 ± 1, 36.29 ± 0.9 and 41.74 ± 1% respectively. Whereas *C. absus*, *F. vulgare*, *M. longifolia*, and *T. cordifolia* showed the highest cell viability percentage (61.32 ± 0.5, 58.99 ± 0.8, 50.27 ± 0.9, and 48.7 ± 0.8) at 400 μg mL ^–1^ concentration as indicated by the initial values. *T. ammi* extracts exhibited maximum cytotoxicity at the lowest extract concentrations of 25–50 μg mL ^–1^. The results are consistent with the reported cytotoxicity of the *T. ammi* extract against the colon carcinoma cell line HCT116 (128) and breast cancer cell line MCF-7 (129).

**FIGURE 5:**
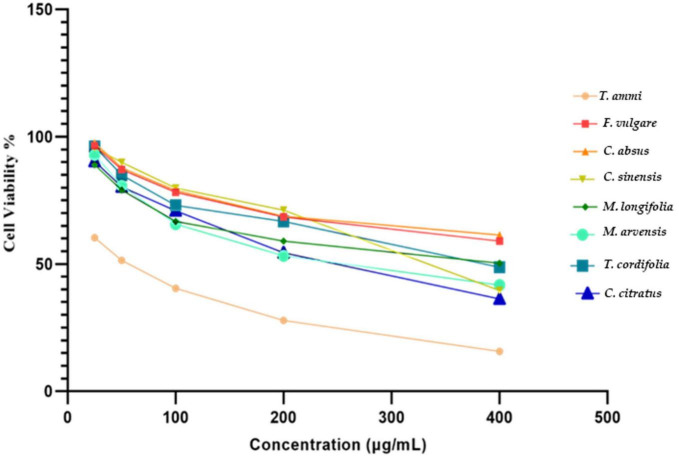
In vitro cytotoxicity of eight different plant extracts against Caco-2 cell line.

The cytotoxicity of the *T. ammi* extract is due to the presence of thymol compounds. Previous investigations documented that thymol has potential cytotoxicity against various cell lines such as monocyte leukemia THP-1 (130), carcinoma HEp-2 (131), and colorectal adenocarcinoma Caco-2 cell line (132). The cell viability rates determined the IC_50_ value of plant extracts at different concentrations. In the Caco-2 cell line, death of more than 50% of the cells occurred from 104 ± 4.6, 111 ± 7.2, and 143 ± 2.0μg mL^–1^ strengths of *M. longifolia*, *M. arvensis*, and *T. ammi* extracts (Table 4 and Figure 5). The results of the present investigation confirm that therapeutic plant extracts can play a significant role in cancer therapy due to their distinct cytotoxic abilities and rich phytochemical composition. Alami et al. (133) recorded 103 plant species belonging to 47 botanical families used by Moroccans to treat cancer. Aristolochia *fontanesii* Boiss. & Reut, Marrubium vulgare L., and *Allium sativum* L. are the most preferred species in Morocco. Medicinal plants used for cancer treatment were classified into four groups: 48 species were used traditionally as anticancer (group a), 41 species were pharmacologically investigated for their anticancer activities (group b), 32 plants with bioactive compounds tested against cancer (group c), and eight plants were clinically investigated for their anticancer effects (group d). Out of 82 plant extracts pharmacologically tested (from plants of group b), only 24 ones show a significant cytotoxic effect. A total of seventy-seven compounds are isolated from plants of group (c). However, only six were clinically evaluated, and most exhibited a beneficial effect on cancerous patients with few side effects.

### 3.6. Antimicrobial activity of MPPE

The eight MPPEs used in the study demonstrated significant bactericidal activity against the microorganisms tested; however, *F. vulgare* exhibited the strongest antimicrobial properties, as shown in Table 5. *S. enterica*, *E. coli*, *B. cereus*, and *S. aureus* are the most common pathogens causing human foodborne illness. Past researchers have also enunciated similar facts that many plant extracts have potent antimicrobial properties, due to which they are effectively being utilized as pharmaceutical agents and food preservatives (134–138). Different concentrations of *F. vulgare* demonstrated powerful antimicrobial activity against the four bacterial strains; however, the extracts of *T. cordifolia* and *M. longifolia* were only effective in preventing the growth of *S. aureus* and *S. enterica*. *M. arvensis* had strong antimicrobial activity (9.3 to 17.3 mm) against *E. coli*, *S. aureus*, and *B. cereus*. Previous studies found that mint leaves exhibited considerable antibacterial activity against *E. coli*, *S. aureus*, and *B. cereus* but did not show any inhibitive activity for *S. enterica* (139, 140).

**TABLE 5:** The antibacterial activity of dietary, medicinal plants extracts.

Plants extract (μg/mL)	*Escherichia coli* (EC)			*Salmonella enterica* (SE)			*Staphylococcus aureus* (SA)			*Bacillus cereus* (BC)			Chloramphenicol			
	400	200	100	400	200	100	400	200	100	400	200	100	EC	SE	SA	BC
*T. ammi*	18.3 ± 1.5^a^	14.6 ± 1.5^d^	9.6 ± 0.5^g^	19.3 ± 1.1^a^	15.3 ± 1.1^b^	10.6 ± 1.1^e^	–	–	–	19 ± 1.0^a^	15 ± 1.0^d^	10.3 ± 1.5^g^	20.2	19.8	19.4	19.1
*F. Vulgare*	15.3 ± 1.5^c^	12 ± 1.0^e^	8.3 ± 0.5^h^	15.6 ± 0.5^b^	13 ± 0.5^c^	9.6 ± 0.5^f^	17.6 ± 0.5^a^	15.3 ± 0.5^c^	10.6 ± 0.5^gh^	17 ± 1.0^b^	11.6 ± 0.5^f^	8 ± 1.0^i^	18.7	18.3	18.3	17.8
*C. absus*	–	–	–	7.7 ± 0.5^h^	7 ± 0.5^i^	6 ± 0.5^j^	11.7 ± 0.5^f^	10.5 ± 0.5^gh^	8.7 ± 05^k^	–	–	–	18.4	18.6	18.9	17
*C. sinensis*	7.3 ± 0.5^i^	–	–	11.6 ± 0.5^d^	9.6 ± 0.5^f^	7.3 ± 0.5^hi^	15.3 ± 0.5^c^	12.6 ± 0.5^e^	10 ± 1.0^hij^	16.6 ± 0.5^bc^	15 ± 1.0^d^	11.6 ± 0.5^f^	17.6	17.6	18.2	17.7
*M. longifolia*	–	–	–	–	–	–	11 ± 1.0d^fg^	8.6 ± 0.5^kl^	6.6 ± 0.5^m^	7.6 ± 0.5^i^	–	–	18.3	18.5	18.2	18.1
*M. arvensis*	17.3 ± 0.5^b^	14.6 ± 0.5^d^	11 ± 1.0^f^	–	–	–	16.3 ± 0.5^b^	13.6 ± 0.5^d^	10.3 ± 0.5^hi^	16 ± 2.0^c^	12.3 ± 1.5^e^	9.3 ± 0.5^h^	20.7	20.9	20.8	21.2
*T. cordifolia*	–	–	–	–	–	–	9 ± 1.0^j^	7.1 ± 0.5^l^	–	7 ± 1.0^j^	–	–	18.7	18.1	18.4	18.3
*C. citratus*	–	–	–	13.3 ± 0.5^c^	10.3 ± 0.5^e^	8.3 ± 0.5^g^	16 ± 1.0^b^	13 ± 1.0^e^	11 ± 1.0^fg^	15 ± 1.0^d^	12.3 ± 0.5^e^	10.6 ± 1.1^g^	17.8	17.5	17.7	17.4

*n* = 3, Data are presented as mean ± SD. Lowercase letters in the same column indicate significant differences at *p* < 0.05; *Escherichia coli (EC)*; *Salmonella enterica* (SE); *Staphylococcus aureus* (SA); *Bacillus cereus* (BC).

Flavonoids, phenols, tannins, alkaloids, and glycoside components of mint are the reason behind its unique bactericidal, antioxidant, cytotoxic, and analgesic characteristics. Another recent research reported that the ethanolic extract of *Mentha* species has potential antimicrobial activity against both G+ and G- bacteria. Still, it was found particularly effective in killing the cells of *A. baumannii.* In recent years, these obstinate and extremely drug-resistant bacteria have emerged as a big threat to the medical fraternity (141).

The diameter of the inhibition zone of *F. vulgare* extracts was recorded between 8 and 17.6 mm against all tested microorganisms. The ethanolic and methanolic extracts of *F. vulgare* had antimicrobial activity ranging between 12 and 14 mm) against *B. cereus* and *E. coli*, which was a little lesser than our results, which might be due to variations in plant preparations (142). The extract of *T. cordifolia* inhibited *S. aureus* (9 mm) and *B. cereus* (7 mm) at a concentration of 400 μg mL^–1^; however, no bactericidal effect was observed at 100 and 200 μg mL^–1^ concentrations.

The extract of *M. longifolia* hindered the activity of *S. aureus* (approximately 6.6–11 mm) at all concentration levels. Still, it only showed inhibition activity for *B. cereus* at a 400 μg mL ^–1^ concentration. Likewise, *C. absus* extracts depicted antimicrobial activity only against *S. aureus* and *S. enterica* at various potencies. Similarly, the methanolic extracts of *C. absus* developed small inhibition zones even at concentrations of 300, 400, and 500 μg mL ^–1^ (30). The extracts of *T. cordifolia* demonstrated antimicrobial activity against *S. aureus* and *B. cereus* only at 400 μg mL ^–1^ concentration.

Moreover, no antimicrobial activity was observed against any bacteria by its extracts at lower concentrations. All concentrations of tea and lemongrass showed moderate inhibitory effects against *B. cereus*, *S. aureus*, and *S. enterica*, but a weaker bactericidal effect was observed against *E. coli*. Similar observations showed that all types of tea, including black and green tea, were restrictive for a wide range of bacteria growth. Tea possesses powerful antioxidants, such as polyphenols and catechins, giving it strong antimicrobial, antimutagenic, and anticarcinogenic properties (143). The strong inhibition activity of tea catechins was found helpful in treating gastric ulcers caused by *H. pylori* (144).

*T. ammi* displayed the greatest bactericidal effect against *S. enterica*, *B. cereus*, and *E. coli* among all plants at different concentrations but did not show any inhibitory effect against *S. aureus*. Ajwain had potent antibacterial and antifungal properties and carminative and antispasmodic characteristics that justify its use as a medicine for digestive problems over the years (145). Similarly, thymol, ρ-cymene, and terpinene contents of *T. ammi* are responsible for the strong antimicrobial and antioxidative properties (146). These functional properties of Ajwain are so powerful that they can be utilized at the industrial level to preserve perishable and high-fat foods. Current research results indicate that plants have variable inhibitory responses to different bacteria depending on their chemical composition and stage of growth. Overall, the inhibitory response of the plants was better against gram-positive pathogens than against gram-negative pathogens; moreover, the antimicrobial activity of the plants was directly dependent on the concentration of extracts and that correlated with recent studies where Cock et al. (147). A total of 149 plant species that are used to treat oral pathogens were identified. The majority of the identified plants are native to southern Africa. However, 23 exotic species used in the healing system of at least one southern African ethnic group were also included. Toothache was the main condition treated with traditional medicines, although plant medicines were also often used to treat oral candidiasis and mouth ulcers. Roots and leaves were most frequently used, and they were generally used either as mouthwashes or by direct application to the affected area. Despite the diverse flora of southern Africa and the ongoing use of plant medicines to treat oral infections, the effects of southern African plants against oral pathogens have been relatively poorly explored. Only 47 of the 149 identified plant species (∼32%) have been verified by laboratory screening. Furthermore, the majority of the plants that have been tested have only been screened against limited panels of oral pathogens. Of the pathogens screened, the effects of the southern African plants against Candida spp. have been the most extensively studied. In contrast, relatively few plant species have been screened against oral bacterial pathogens, except against *Streptococcus mutans*.

### 3.7. Principal component analysis, correlation coefficient, and cluster analysis

**FIGURE 6:**
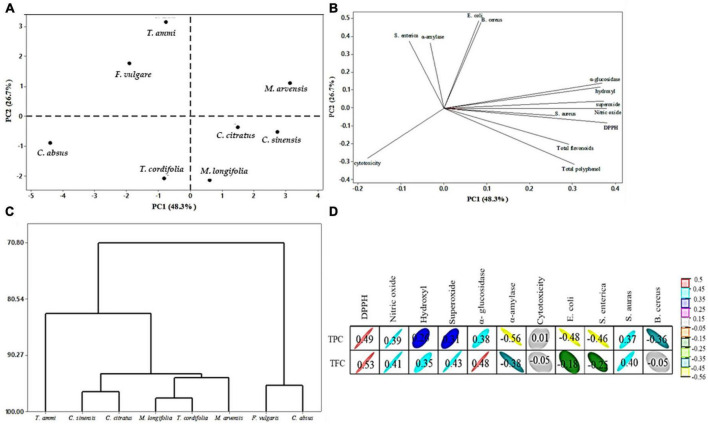
Principal component analysis (PCA) of eight different plant extracts; **(A)** location of plant extracts (treatment), **(B)** location of variables: TPC, TFC, four different antioxidant activity (DPPH, NO, OH, O2^–^), antimicrobial activity, enzymes inhibition, and cytotoxicity (parameters), **(C)** cluster analysis of eight plant extracts, **(D)** pearson correlation coefficient (R) between bioactive compound and biological activity.

Principal Component Analysis (PCA) was used to summarize similarities or differences among the results of eight different plant extracts considering 13 variables, including total polyphenols, total flavonoids, cytotoxicities, antioxidant activities (DPPH, NO, O_2_, OH), inhibition of enzymes, and antimicrobial activities (EC, SE, SA, BC). Data in the score plot (Figure 6A) shows eight MPPEs, while data in the loading plot (Figure 6B) shows the parameters/variables of the score. The sum of the first two principal components, PC1, and PC2, accounted for 75% of the total variation between plant extracts. In contrast, the central component (PC1) accounted for 48.3%, while the principal component (PC2) accounted for 26.7% variability in the dataset.

The PCA score plot shows the clear grouping of plant extracts, making visible the clear difference between *F. vulgare*, *T. ammi*, and *M. arvensis* (first and second quarter of PC2 positive) and *C. absus*, *T. cordifolia*, *M. longifolia*, *C. citratus*, and *C. sinensis* (third and fourth quarter of PC2 negative). The result suggested different plant extracts have different antioxidant, antimicrobial, antidiabetic, and cytotoxic activities. *M. arvensis* is the only plant in the first quarter with the highest contribution to PC2. The extract of *T. ammi* is at the highest position in the second quarter of PC2, presenting a high qualitative difference from other treatments. It showed that the extract of *T. ammi* has potential antimicrobial activity (*E. coli, B. cereus*, and *S. enterica*) and α-amylase inhibition activity (Figure 6B). The extracts of *F. vulgare* and *T. ammi* were in the same quarter of PC2, from which it can be inferred that there is the least significant difference between them. Similarly, *C. absus* and *T. cordifolia* extracts were in the same quarter. Moreover, the extracts of *Cymbopogon citratus* and *Camellia sinensis* were in the same quarter close to the *M. longifolia* extract, indicating that there is a slight disparity between these extracts; the result also depicted the potential antioxidant activity (DPPH, hydroxyl, NO, and superoxide), antimicrobial activity (*S. aureus*) and α-glucosidase inhibition activity of these plants.

The PC1 is strongly correlated with the different variables in Figure 6B. PC1 increases with increasing total flavonoid, total polyphenol, and antioxidant activity, while PC2 increases with increasing cytotoxicity. The plot also indicated that total polyphenol, total flavonoid, antioxidant, antimicrobial, and enzymes had a positive correlation, while cytotoxicity negatively correlated with plant functional properties. Statistically, the correlation coefficient between bioactive compounds and biological activity is shown. TPC had a significant positive correlation (R = 0.49 to 0.37) with DPPH, NO, α-glucosidase inhibition, and antimicrobial activity *(S. aureus*). In contrast, it had a moderate correlation (R = 0.26 to 0.01) with hydroxyl radical, superoxide radical scavenging activity, and cytotoxicity Figure 6D.

According to Khan et al. (148), total polyphenol content has a (+R) with antioxidant activity but a (−R) correlation with α-amylase and α-glucosidase; However, our results showed a (+R) correlation with α-glucosidase and a (−R) correlation with -amylase, which may be due to differences in the chemical composition of plant extracts. On the contrary, TPC had a negative correlation (R = −0.56 to −0.36) with α-amylase and antimicrobial activity (*E. coli*, *S. enterica*, and *B. cereus*).

TFC exhibited a negative correlation with cytotoxicity, while TPC had a positive correlation with cytotoxicity, suggesting that phenolic compounds modulate the response to cytotoxicity, which was also reported by Fidelis et al. (149); Thiruvengadam et al. (150). The cluster analysis showed that the PCA divided the eight plant extracts into three groups, Figure 6C. The first group consisted of *T. ammi*, and the second group was divided into two subgroups, *C. sinensis* and *C. citratus*, which were present in one subgroup. In comparison, another subgroup was divided into two subgroups, *T. cordifolia* and *M. longifolia* were present in a subgroup, and *M. arvensis* was in another subgroup. Lastly, *F. vulgare* and *C. absus* made a third group.

## 4. Conclusions

It can be concluded from the present study that indigenous medicinal plants are rich in polyphenols that are characterized by stability, bioavailability, and bioactivity than polyphenolic monomers. They excreted unique antioxidant, anti-inflammatory, antidiabetic, lipolytic, antimicrobial, and cancer curative abilities. The free radical scavenging properties of MPPE can be employed in the food industry to preserve the aroma, taste, and flavor and extend foods’ lifetime through natural substances. Indigenous floral species should be thoroughly researched for new biologically active compounds and potential cures against chronic diseases and resistant microbes. Wild dietary and medicinal plants have also gained significance in modern times due to their cost effectiveness, safety, multifunctionality, and virtually no side effects compared to synthetic medicines. The researchers are now trying to discover both individual and combined effects of plant-derived functional compounds on the prognosis of various diseases. In conclusion, the present study affirmed the benefits and effectiveness of ancestral use of native plant species to treat burns, wounds, infections, and various bodily diseases, besides potential usage in human nutrition.

## Data availability statement

The raw data supporting the conclusions of this article will be made available by the authors, without undue reservation.

## Author contributions

AM, SS, SK, and ME-S: conceptualization, software, validation, investigation, resources, writing—original draft preparation, visualization, supervision, and project administration. AM, SS, SK, MFA, and ME-S: methodology, and data curation. AM, SS, SK, DA-Q, MAlh, HA, NB, MAlj, KM, and ME-S: formal analysis. AM, SS, SK, DA-Q, MAlh, MASA, HA, NB, MH, MAlj, KM, and ME-S: writing—review and editing. DA-Q, MAlh, MASA, HA, NB, MAlj, and KM: funding acquisition. All authors have read and agreed to the published version of the manuscript.
